# Structured carbon foam derived from waste biomass: application to endocrine disruptor adsorption

**DOI:** 10.1007/s11356-019-06302-8

**Published:** 2019-10-19

**Authors:** Mohamed Zbair, Satu Ojala, Hamza Khallok, Kaisu Ainassaari, Zouhair El Assal, Zineb Hatim, Riitta L. Keiski, Mohamed Bensitel, Rachid Brahmi

**Affiliations:** 1 Laboratory of Catalysis and Corrosion of Materials (LCCM), Department of Chemistry, Faculty of Sciences, University of Chouaïb Doukkali, BP 20, 24000 El Jadida, Morocco; 2 Faculty of Technology, Environmental and Chemical Engineering, University of Oulu, P. O. Box 4300, FI-90014 Oulu, Finland; 3 Team of Energy, Materials, and Environment, Department of Chemistry, Faculty of Sciences, University Chouaïb Doukkali, El Jadida, Morocco; 4 Laboratory of Coordination and Analytical Chemistry (LCCA), University Chouaïb Doukkali, El Jadida, Morocco

**Keywords:** Endocrine disruptor, Bisphenol A, Adsorption, Carbon foam, Regeneration, Water purification

## Abstract

In this paper, a novel structured carbon foam has been prepared from argan nut shell (ANS) was developed and applied in bisphenol A (BPA) removal from water. The results showed that the prepared carbon foam remove 93% of BPA (60 mg/L). The BPA equilibrium data obeyed the Liu isotherm, displaying a maximum uptake capacity of 323.0 mg/g at 20 °C. The calculated free enthalpy change (*∆H*° = − 4.8 kJ/mol) indicated the existence of physical adsorption between BPA and carbon foam. Avrami kinetic model was able to explain the experimental results. From the regeneration tests, we conclude that the prepared carbon foam has a good potential to be used as an economic and efficient adsorbent for BPA removal from contaminated water. Based on these results and the fact that the developed structured carbon foam is very easy to separate from treated water, it can serve as an interesting material for real water treatment applications.

**Electronic supplementary material:**

The online version of this article (10.1007/s11356-019-06302-8) contains supplementary material, which is available to authorized users.

## Introduction

Endocrine-disrupting substances are chemical compounds or mixtures of compounds that alter endocrine system functions of organisms and, as a result, cause adverse effects on the health of living organisms, their offspring, or part of the population (Vos et al. 2000; López-Ramón et al. 2019). Endocrine disruptors (EDs) can, for example, inhibit or stimulate the secretion of hormones, interfere with the hormone receptor (agonist or antagonist effect), and modify the metabolism of these enzymes (Mills and Chichester 2005). The disturbances are caused after prolonged exposure to these compounds even at low concentrations. A large number of pollutants have been identified as EDs. These comprise organic compounds such as alkyphenols, chlorinated pesticides, herbicides, and drug compounds (Singleton and Khan 2003). The type of disturbance caused by these compounds is related to the structural similarity between the compound and the hormone.

Despite the existing water treatment, certain pharmaceuticals and EDs end up to the aquatic environment. Numerous endocrine-disrupting substances or metabolites from their biodegradation are found in the effluents and sewage sludge from wastewater treatment plants (Auriol et al. 2006; Tan et al. 2007). Among these endocrine-disrupting substances found, nonylphenol (NP), bisphenol A (BPA), and triclosan (TCS) are the absolute most regularly identified organic xenobiotics in wastewater treatment areas (Kolpin et al. 2002; Boyd et al. 2003, 2004). These substances are distinguished by the existence of a phenolic group in their chemical structure. The OH-group can improve the adsorption of these substances on materials that contain functional surface OH-groups (Zbair et al., 2018a, b, d).

This research focuses on the elimination of a phenolic EDs, bisphenol A (BPA), from aqueous solution. BPA is acutely toxic to the aquatic organisms from 1 to 10 mg/L concentration range for both fresh water and marine environment (Kang et al. 2006; Aravind et al. 2019). BPA has been detected in several types of water at different concentrations. For example, concentration of 17.2 mg/L has been observed in in hazardous waste landfill leachate (Yamamoto et al. 2001), 12 μg/L in stream water (Liu et al. 2009a), and 3.5–59.8 ng/L in drinking water (Santhi et al. 2012). The solubility of BPA in water is 300 mg/L in normal conditions (Shareef et al. 2006).

Numerous materials such as activated carbons (Asada et al. 2004; Liu et al. 2009b; Zbair et al. 2018a), CoFe_2_O_4_/activated carbon (Li et al. 2014), hybrid material (phenyl-mesoporous silica) (Bhatnagar and Anastopoulos 2017), graphene (Wang et al. 2017), and Fe/AC (Arampatzidou et al. 2018) have been propositioned for removal of BPA. Porous adsorbents are promising materials for adsorption of organic pollutants, because of good physiochemical stability, large specific surface area, and well-developed porosity with high pore volume (Agrawal et al. 2016; Jana et al. 2016; Zbair et al. 2018b). It is known that porous carbon materials are thermally stable, chemically inert, and low-cost and display high adsorption capacity for organic pollutants. The earlier research shows that carbon foam is efficient in the removal of copper, arsenic (V), and lead (Burke et al. 2013; Lee et al. 2015; Agrawal et al. 2016). Therefore, in the current work, we decided to apply carbon foam, prepared from waste biomass (argan nut shell) in BPA removal. The aim was to develop a material that can be easily separated from the treated water and increase surface contact solid/liquid. The chemical composition and surface characteristics of the carbon foam were examined. Batch adsorption experiments were conducted to evaluate the BPA uptake, adsorption mechanism, and possibility to regenerate the carbon foam.

## Experimental section

### Preparation of carbon foam

The argan nut shells (ANS) were collected from the Southwestern Morocco (Taroudant city). First, the raw material was washed with tap water and distilled water. The washed ANS was oven dried at 105 °C for 12 h. After this, the raw material was ground and sieved to a particle size of 200 μm. Then the ANS was pyrolyzed at 600 °C for 2 h under a nitrogen flow (100 mL/min). The employed technique, to produce carbon foam (CF), initially involves the preparation of aqueous slurry from a mixture of pyrolyzed ANS, dispersing agent, structuring agent, and water. Sucrose was used as the both—as the dispersant and the pore promoter (Pradhan and Bhargava 2004). At first, 2.1 g of sucrose was mixed with 10 mL of double distilled water for 1 h. Then, 3 g of pyrolyzed ANS was added step by step to sucrose gel under stirring at 500 rpm until reaching a concentration of 70 wt-% relative to the pyrolyzed ANS. The resulting slurry was stirred for 24 h for homogenization. Then, the slurry was poured into a cylindrical silicone mold and dried firstly at 80 °C for 1 h and then at 105 °C for 24 h to eliminate the surface water and initialize the sucrose consolidation. The molded sample was pyrolyzed under a nitrogen flow of 50 mL/min from the room temperature to 900 °C according to the following cycle: first heating was done with a rate of 0.5 °C/min from room temperature to 500 °C to eliminate sucrose totally (Das 2001). Then the sample was kept at a constant temperature for 1 h followed by heating with a rate of 5 °C/min up to 900 °C, where the temperature was kept constant for 5 h. Finally, cooling was done with the rate of 2 °C/min back to the room temperature.

### Characterization

The N_2_ adsorption-desorption isotherm of carbon foam was examined using a Micromeritics ASAP 2020 instrument (Micrometrics, Norcross, GA, USA) to determine surface area, pore volume, and pore size distribution. The morphological characteristics of carbon foam were analyzed using a field emission scanning electron microscope ZEISS ULTRA PLUS (ZEISS, Oberkochen, Germany) equipped with an energy-dispersive X-ray spectrometer (EDS) at an accelerating voltage of 15.0 kV. The acidity and basicity measurements of carbon foam were done using temperature-programmed desorption (TPD) of NH_3_ and CO_2_ with an AutoChem II 2920 (Micromeritics, Norcross, GA, USA) device. Prior to NH_3_-TPD analysis, the carbon foam (about 100 mg) was pre-treated with helium (He) at 700 °C for 30 min. Then, carbon foam was cooled to 100 °C followed by adsorption of 15% NH_3_ in He (at 100 °C) for 60 min and flushing with He for 30 min, in order to eliminate the physiosorbed NH_3_. The NH_3_ desorption was carried out from 100 to 700 °C including 10-min constant temperature dwell at the final temperature. The flow rate used was 50 cm^3^/min and temperature rise was 10 °C/min during the NH_3_-TPD analysis. After NH_3_-TPD analysis, the carbon foam was cooled to room temperature prior CO_2_-TPD. The sample was pre-treated with H_2_ (30 cm^3^/min) from room temperature to 500 °C with 10 °C/min for 30 min, then cooled to 50 °C and flushed with Ar with 50 cm^3^/min for 5 min. The adsorption of 5%CO_2_/Ar (50 cm^3^/min) was completed at 50 °C for 60 min then the physiosorbed CO_2_ was flushed with Ar (50 cm^3^/min) for 60 min. The thermodesorption of CO_2_ was carried out under Ar flow (50 cm^3^/min) from 50 to 700 °C, where temperature was left constant during 10 min. The concentrations of desorbed NH_3_ and CO_2_ were analyzed by a thermal conductivity detector (TCD) and the total acidity and basicity of carbon foam were determined by integration of the peak area between 100–700 °C and 40–700 °C, respectively. The PZC (point of zero charge) of carbon foam was determined using the pH drift method. The carbon foam was mixed with 60 mL of 0.01 M NaCl solution. The pH of the starting solutions (2.0 to 12.0) was adjusted using HCl and NaOH. After 24 h, the final pH was measured. The functional surface groups of carbon foam were determined using a Fourier transformed infrared spectroscopy (FTIR–8400S, Shimadzu, Japan).

### Batch adsorption experiments

A stock solution of BPA (100 ppm) was prepared by dissolving the BPA (Alfa Aesar, 97%) in distillated water. Then, the BPA solutions of desired concentrations were prepared by successive dilutions from their respective stock solution. The effect of solution pH on BPA (15 mg/L) removal was examined at various initial pH values (2.0–12.0) for 2 h, the solutions were adjusted by using 1 M HCl or 1 M NaOH. The adsorption kinetics was studied using two distinctive starting BPA concentrations, 15.0 mg/L and 60.0 mg/L. The adsorption isotherm was examined for BPA concentrations of 10–100 mg/L at different temperatures (20 °C, 30 °C, and 40 °C). After pre-decided times, the samples were taken to assess the remaining BPA concentration in the solution. The BPA concentration was determined using a UV-visible spectrophotometry (SHIMADZU 2450-UV/VIS, JAPAN) at the maximum absorbance wavelength (274 nm). All batch adsorption experiments of BPA were carried out with a mass of 50 mg of carbon foam in 200 mL of BPA solution.

### Regeneration

The BPA-laden carbon foam was regenerated using 30 mL of ethanol (Sigma-Aldrich, 99.8%). Ethanol and the used carbon foam were stirred at room temperature for 4 h. At that point, the carbon foam was recovered by filtration and dried at 80 °C. Adsorption and regeneration cycles were repeated 5 times.

### Modeling

The model parameters of adsorption kinetics and adsorption isotherm were calculated by a non-linear regression method. All equations and models used in this work are presented in Table 1S.

**Table 1:** Kinetic and equilibrium adsorption parameters

	15 mg/L	60 mg/L
Pseudo-first-order		
*Q*_e_,cal (mg/g)	*54.77*	*218.49*
*K*_1_ (min^−1^)	0.452	0.220
*t*_1/2_ (min)	1.533	3.150
*t*_0.95_ (min)	7.050	13.450
*R*^2^	*0.990*	*0.972*
SD (mg/g)	*0.429*	*3.034*
Pseudo-second-order		
*Q*_e_,cal (mg/g)	*55.63*	*226.66*
*K*_2_ (g/mg^−1^ min^−1^)	0.026	0.001
*t*_1/2_ (min)	0.782	2.605
*t*_0.95_ (min)	11.51	41.67
*R*^2^	*0.996*	*0.995*
SD (mg/g)	*0.368*	*1.580*
Avrami-fractional-order		
*K*_AV_ (min^−1^)	0.281	2.111
*Q*_e_,cal (mg/g)	*55.11*	*222.36*
*n*_AV_	1.064	0.055
*t*_1/2_ (min)	*2.251*	*5.573*
*t*_0.95_ (min)	*9.961*	*24.217*
*R*^2^	*0.999*	*0.999*
SD (mg/g)	*0.340*	*1.157*

## Results and discussion

### Characterization of carbon foam

**Fig. 1:**
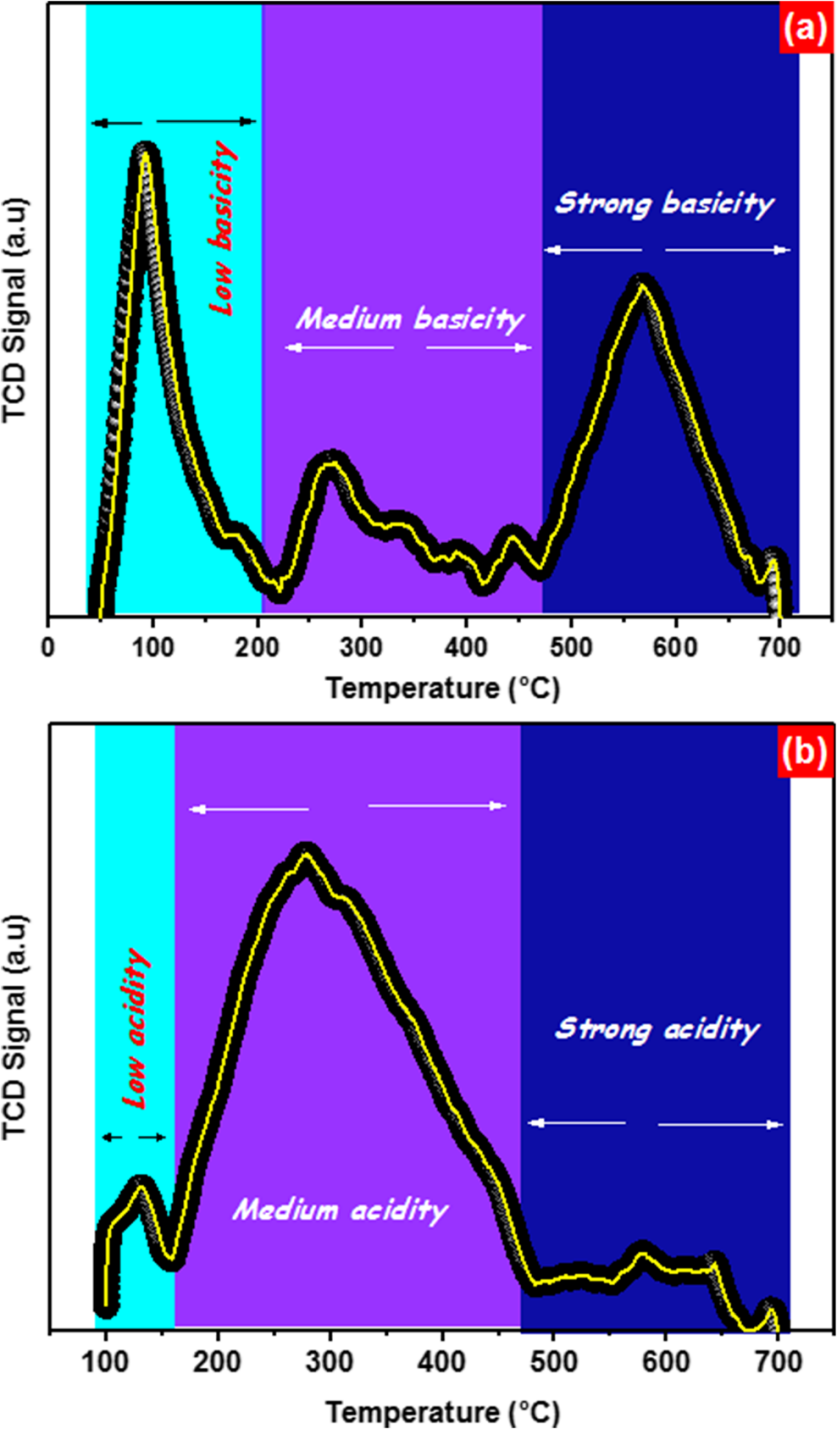
**a** Temperature-programmed desorption (TPD) of CO_2_ and **b** temperature-programmed desorption (TPD) of NH_3_

The CO_2_-TPD profiles of carbon foam exhibited the peaks in the temperature ranges of 50–200, 200–460, and upper than 460 °C that are signifying desorption of CO_2_ from the weak, medium, and strong basic sites (Cho et al. 2005; El Assal et al. 2018). The carbonaceous materials constructed from biomass have predictably somewhat high amount of hydroxyl groups (–OH), which can adsorb CO_2_ (Meng et al. 2006). As appeared in Fig. 1 a, the peak between 50 and 200 °C is linked to the existence of the feeble amount of weak basic sites in carbon foam. Also, the peaks observed between ∼ 200 and 460 °C suggest the presence of medium basic sites. The strong basic sites were detected at a temperature above 460 °C. The NH_3_-TPD profiles of carbon foam are shown in Fig. 1b. Low, medium, and strong acid sites are observed in the temperature ranges of 100–170, 170–480, and > 480 °C, respectively (Arena et al. 1998; El Assal et al. 2017). The maxima of the first and second peak were positioned at 130 and 279 °C. These peaks are analogous to the presence of weak and medium acid sites in carbon foam. Besides, above 480 °C, carbon foam showed only a very small amount of strong acid sites. The quantification showed that carbon foam contains 1.538 mmol/g of acid sites and 0.545 mmol/g of basic sites in total.

**Fig. 2:**
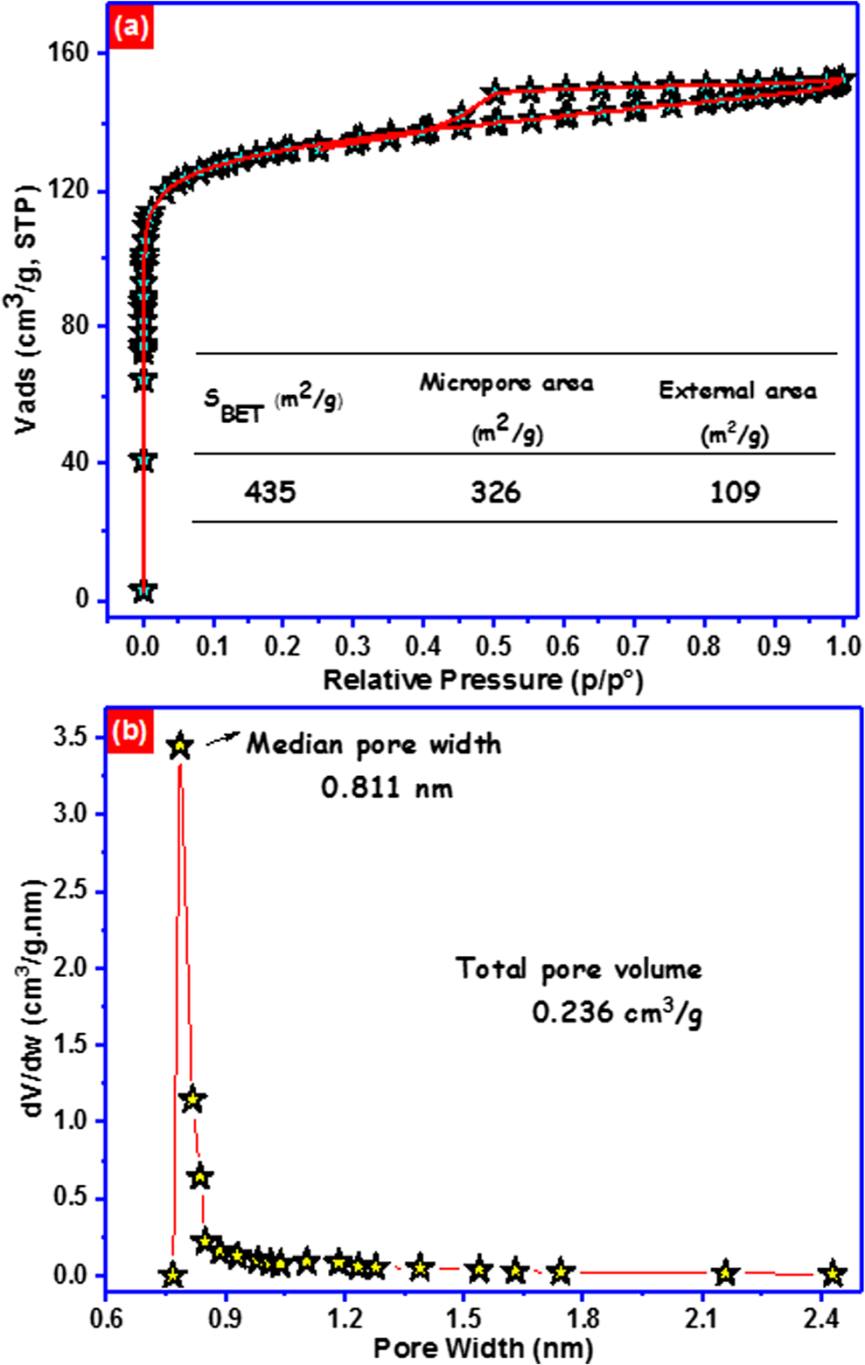
**a** N_2_-sorption isotherm. **b** Distribution pores width of carbon foam

To inspect the pore characteristics of the carbon foam, N_2_ adsorption/desorption isotherms are presented in Fig. 2a. The N_2_ isotherm of carbon foam is a blend of type I and type IV isotherms, enlightening microporous and mesoporous textural properties. The sharp increment in the adsorption volume at very low relative pressure (Long et al. 2015) demonstrates the presence of micropores in the carbon foam. Besides, the type H2 hysteresis loop (Rouquerol et al. 1999), which outlines the interconnected networks of pores of diverse sizes and forms, is observed. The carbon foam has a specific surface area (*S*_BET_) of 435 m^2^/g and a total pore volume of 0.236 cm^3^/g. The distribution of pore width based on the Horvath-Kawazoe method indicates that the average pore width is about 0.811 nm (Fig. 2b).

**Fig. 3:**
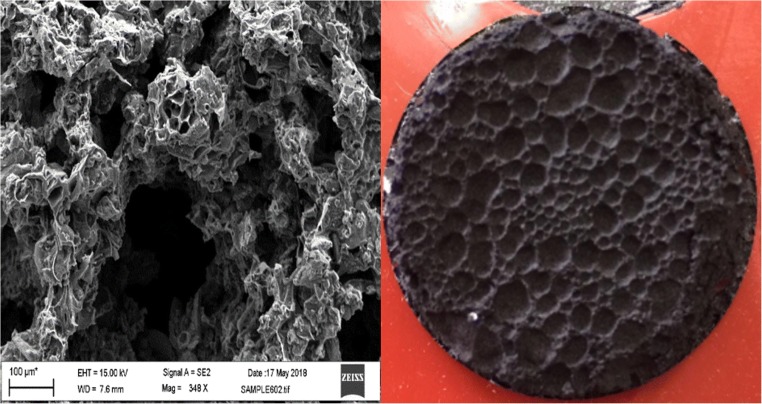
FESEM image and photo of the carbon foam

The SEM images of the carbon foam show a hierarchical pore structure with different shapes and sizes of pores from macropores to micropores (Fig. 3). The observed pores are irregular, and the walls between the larger pores (~ 100 μm) contain smaller pores with around 10-μm diameter.The developed porosity of carbon foam can facilitate the adsorption of organic pollutants due to an increased residence time and turbulence inside the foam. The EDS mappings of the carbon foam (Fig. 1S) indicate the presence of six main elements: C (84.27 %), O (12.34%), Na (0.48%), S (1.22%), and K (0.48%).

### Adsorption kinetics

**Fig. 4:**
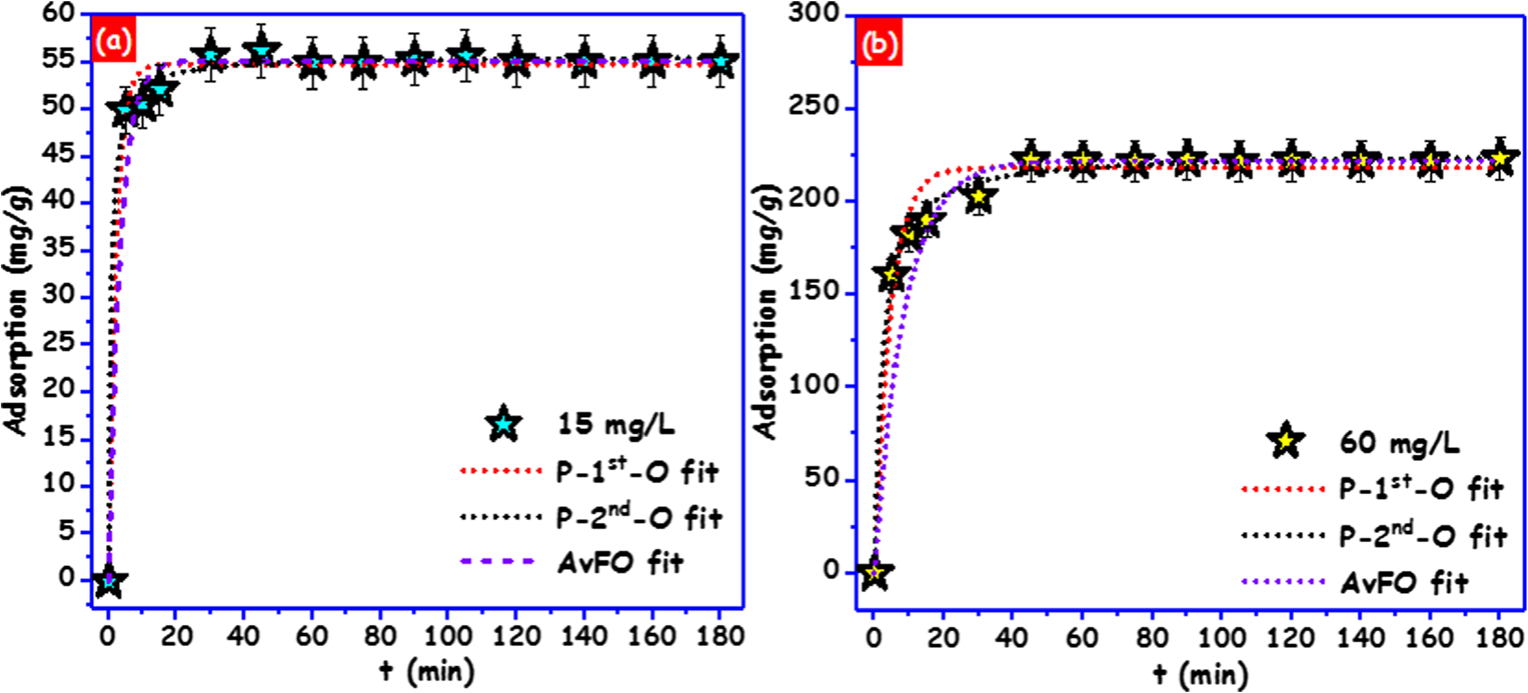
Adsorption kinetics of BPA onto carbon foam fitted by pseudo-first order (P-1^st^-O), pseudo-second order (P-2^nd^-O), and Avrami fractional-order (AvFO) kinetic models for 15 mg/L and 60 mg/L of BPA

To explain the kinetics of BPA adsorption on the carbon foam, three kinetic models were tested (Fig. 4): the Avrami fractional-order kinetics (AvFO), the pseudo-first-order kinetics (P-1^st^-O), and the pseudo-second-order kinetics (P-2^nd^-O) (Lima et al. 2015; Tran et al. 2017; Kasperiski et al. 2018). The correctness of the fitting was evaluated by using *R*^2^ and standard deviation (SD). Higher *R*^2^ and lower SD values specify the inconsistency between the experimental and the theoretical adsorption capacity (Qcal) values. In this study (Table 1), from the three tested kinetic models, the AvFO kinetic model attained the highest *R*^2^ values of 0.999 for the both used concentrations. It also showed the lowest SD of 0.340 mg/g and 1.157 mg/g, for 15 mg/L and 60 mg/L initial BPA concentration, respectively. This designates that the Qcal estimated by the AvFO kinetic equation is able to explain best the BPA adsorption on the carbon foam. The kinetic rate constant of the models used in this work has different units (Thue et al. 2016), which makes their direct comparison problematic. Hence, the times to reach 50% (*t*_0.5_) and 95 % (t_0.95_) adsorption of BPA onto the carbon foam at the equilibrium (*Q*_e_) were interpolated from the fitted kinetic curve according to ref. (Kasperiski et al. 2018). Based on the AvFO kinetic model, the values of *t*_0.5_ were 2.251 min and 5.573 min for the initial BPA concentrations of 15.0 mg/L and 60.0 mg/L, respectively. The times to reach 95% (*t*_0.95_) adsorption, (*Q*_e_) were 9.961 min and 24.217 min for 15 mg/L and 60 mg/L of BPA initial concentrations, respectively. As a result, the adsorption time for further experiments was fixed at 60 min in order to guarantee that at higher BPA concentrations maximum adsorption is reached.

### Adsorption isotherms

**Fig. 5:**
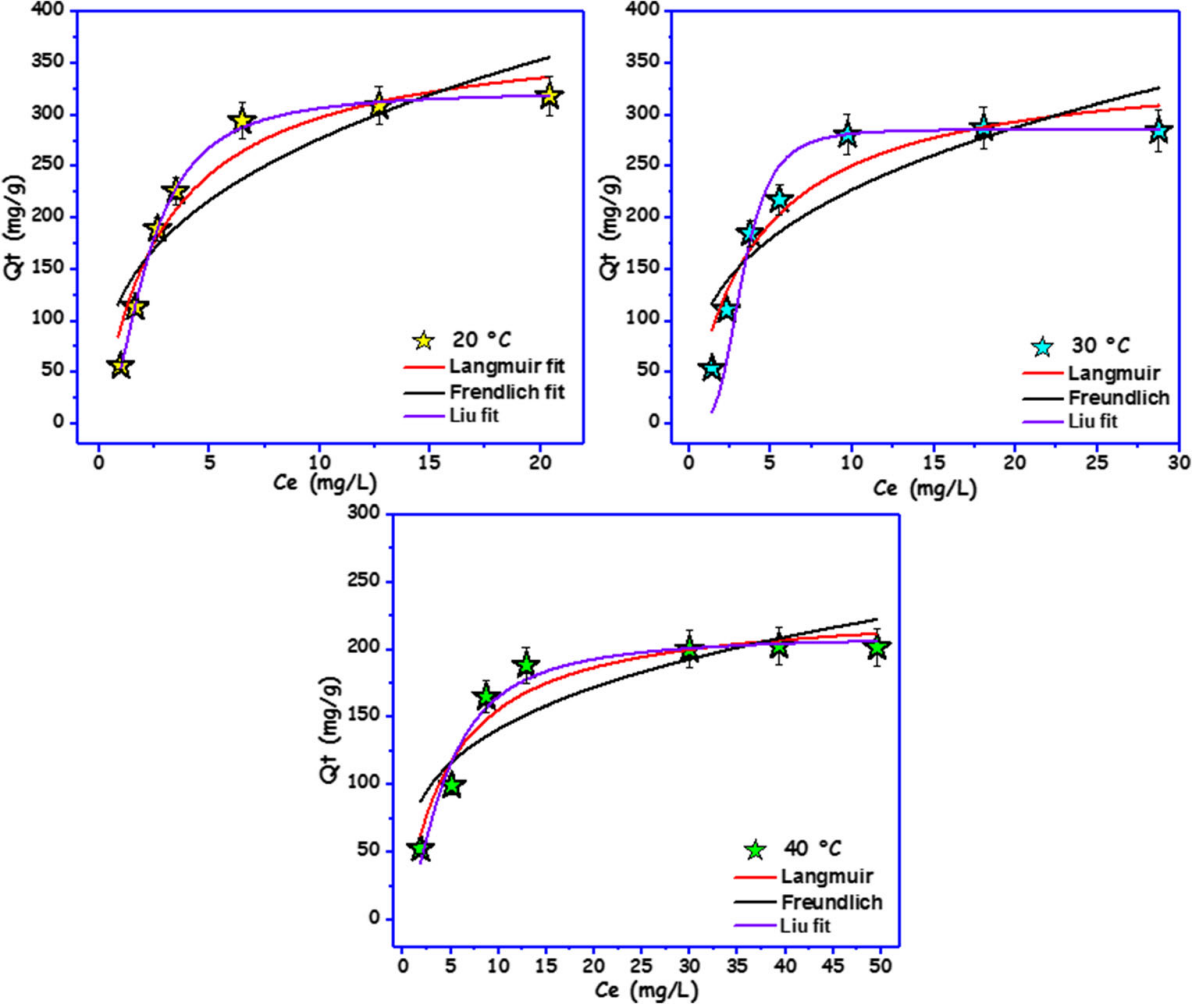
Adsorption isotherm of BPA (*C*_0_ 10–100 mg/L) onto carbon foam at various temperatures (20 °C, 30 °C, and 40 °C) and fitted by Langmuir, Freundlich, and Liu models

The utilization of adsorption isotherms helps to explain the mechanism of adsorption (Lima et al. 2015; Kasperiski et al. 2018). There exists a number of isotherm equations used widely to explain the experimental adsorption data (Lima et al. 2015; Tran et al. 2017). Among the available isotherms, for this study, Langmuir, Freundlich, and Liu’s models were selected. Figure 5 presents the adsorption isotherms of BPA on the carbon foam at 20 °C, 30 °C, and 40 °C. Based on the SD and *R*^2^, the Liu model was the best model to explain BPA adsorption onto the carbon foam (Table 2). The Liu model showed that the SD values varied from 1.117 to 10.639, and the *R*^2^ values ranged from 0.970 to 0.999. In the Liu model, it is assumed that the adsorption sites do not have the same adsorption energy with each other. Earlier, we have shown that the carbon foam has different types of surface functional groups, which is in agreement with the assumption of the Liu model assumption (Dias Lima et al. 2007; Prola et al. 2013).

**Table 2:** Isotherm parameters for the adsorption of BPA onto the carbon foam

Temperature	20 °C	30 °C	40 °C
Langmuir isotherm			
*Q*_L_ (mg/g)	*386.0*	*353.0*	*223.0*
*K*_L_ (L/mg)	0.339	0.246	0.201
*R*^2^	*0.943*	*0.911*	*0.929*
SD (mg/g)	29.280	32.515	14.433
Freundlich isotherm			
*K*_F_ (mg/g) (L/mg)^1/*n*^	123.0	104.0	74.0
*n*	2.848	2.939	3.544
*R*^2^	*0.792*	*0.740*	*0.761*
SD (mg/g)	23.573	24.930	18.027
Liu isotherm			
*Q*_Li_ (mg/g)	*323.0*	*287.0*	*211.0*
kg (L/mg)	0.455	0.304	0.226
*n*_L_	1.950	3.708	1.567
*R*^2^	*0.998*	*0.999*	*0.970*
SD (mg/g)	*3.558*	*1.117*	*10.639*

According to Table 2 and Fig. 5, it is possible to verify that the adsorption capacity of the carbon foam decreases with increasing temperature. The variation of temperature from 20 to 40 °C weakened the adsorption capacity of the carbon foam. The maximum adsorption (*Q*_Li_) of the carbon foam for BPA was the following: 323.0 mg/g at 20 °C, less than 287.0 mg/g at 30 °C and less than 211.0 mg/g at 40 °C. Moreover, the values of the Liu equilibrium constant (*K*_g_) decreased with increasing temperature indicating exothermic adsorption.

In order to compare the uptake capacity of the carbon foam in the removal of the BPA from aqueous solutions, a comparison of BPA adsorption capacities of other adsorbents is presented in Table 3. It can be noted that the carbon foam showed respectable adsorption capacity when compared to the other type of materials. The carbon foam exhibited a high *Q*_max_ among the 11 adsorbents inspected, which supports the exceptionally good performance of the carbon foam.

**Table 3:** Maximum adsorption capacities of BPA by different adsorbents

Adsorbents	Solid/liquid ratio	*Q*_max_ (mg/g)	References
Mesoporous carbon (soft templated)	0.01 mg/100 mL	156.0	(Libbrecht et al. 2015)
Graphene	0.05 g/20 mL	94.06	(Bele et al. 2016)
Graphite oxide	0.05 g/20 mL	17.27	(Bele et al. 2016)
Carbon nanotubes (CNTs)	5 g/L	46.18	(Li et al. 2015)
Montmorillonite modified with DDDMA	(0.2–0.3 g)/40 mL	256.4	(Park et al. 2014)
Hydrophobic zeolite	0.5 g/L	111.11	(Tsai et al. 2006a)
Coconut-based activated carbon	0.5 g/L	263.2	(Tsai et al. 2006b)
Graphene	0.01 g/100 mL	181.8	(Xu et al. 2012)
Activated carbon	20 mg/100 mL	137	(Qin et al. 2015)
ANS@H2O-120	0.01 g/200 mL	1408	(Zbair et al. 2018b)
Modified biomass–based carbon	5 g/L	41.5	(Juhola et al. 2018)
Commercial activated carbon	0.01 g/100 mL	307	(Libbrecht et al. 2015)
Carbon foam	0.05 g/200 mL	323.0	*This work*

### Effect of pH on BPA adsorption

**Fig.6:**
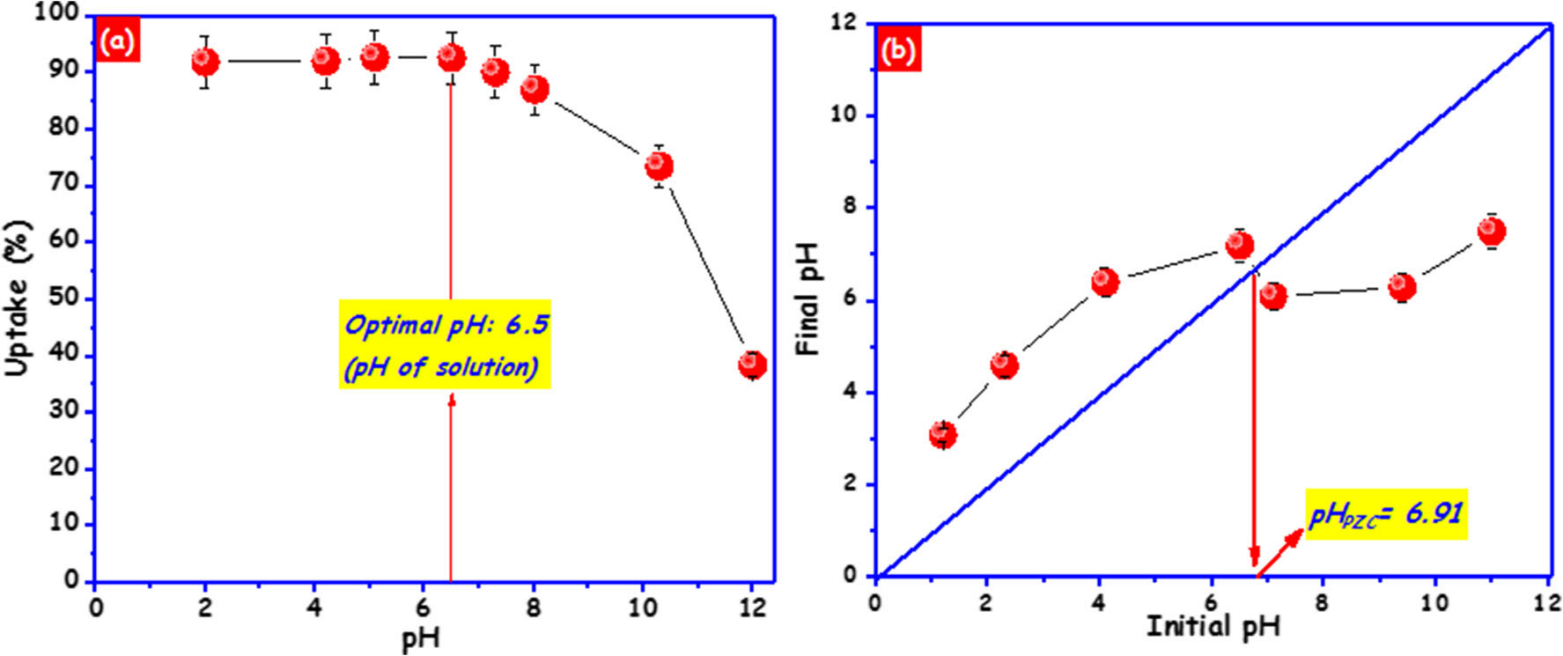
**a** Effect of pH on BPA adsorption (*C*_0_ 15 mg/L; 50 mg of carbon foam; 20 °C; pH 2–12; 2 h). **b** Determination of point of zero charge (pH_PZC_) of carbon foam

The solution pH mostly affects the degree of ionization of BPA and the surface properties (surface charges) of the carbon foam consequently affecting the adsorption of BPA (Zbair et al. 2018a; López-Ramón et al. 2019). In this experiment, the influence of solution pH in the range of 2.0–12.0 on the uptake of BPA (15 mg/L) was examined at room temperature. The outcomes demonstrated that when the pH is < 8.0 (Fig. 6a), the uptake of BPA was not influenced by the initial pH of the medium. Figure 6b shows that the pH_PZC_ of carbon foam is 6.91. At this pH level of a solution (6.5), the net surface charge of carbon foam is near to zero, and the π–π dispersion interaction takes place. The π−π interaction has been applied to describe the adsorption mechanism of the organic molecules such as BPA with benzene rings or C=C double bonds adsorbed on the carbonaceous material (Ahsan et al. 2018). In the case of carbon foam, the interaction between the π electrons of the benzene rings of BPA and carbon foam occurs through the π−π electron coupling mechanism. However, when the solution pH is 8.0 and above, the surface charge of carbon foam is negative and bisphenol A molecules are ionized to their mono- (BPA^−^) and divalent (BPA^2−^) anions (Bautista-Toledo et al. 2005). Therefore, the repulsive electrostatic interaction is strengthened. This was the main cause to the low removal efficiency of BPA onto carbon foam at pH > 8.0. These outcomes are consistent with earlier investigations (Tsai et al. 2006a; Soni and Padmaja 2014; Zbair et al. 2018a).

### Regeneration

**Fig. 7:**
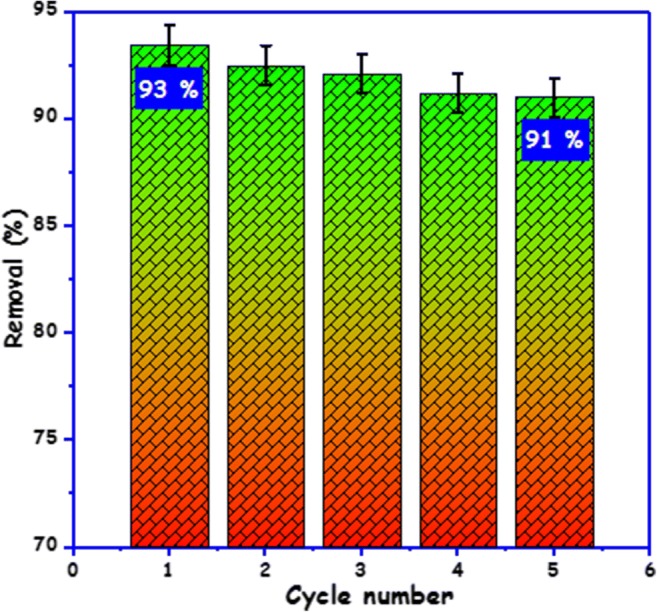
Regeneration using ethanol and reuse of carbon foam for adsorption of BPA

Regeneration studies are critical to evaluate the recyclability of adsorbents for practical applications owing to important economic and ecological demands for sustainability (Ahsan et al. 2018). Consequently, we decided to wash the used carbon foam with ethanol to regenerate it and to find out information concerning the longer-term use of the carbon foam. The regenerated carbon foam was able to keep almost the same adsorption capacity after the second regeneration and stayed at 91 % after 5 cycles (Fig. 7) indicating that the carbon foam has a decent lifetime.

### Proposed adsorption mechanism

**Fig. 8:**
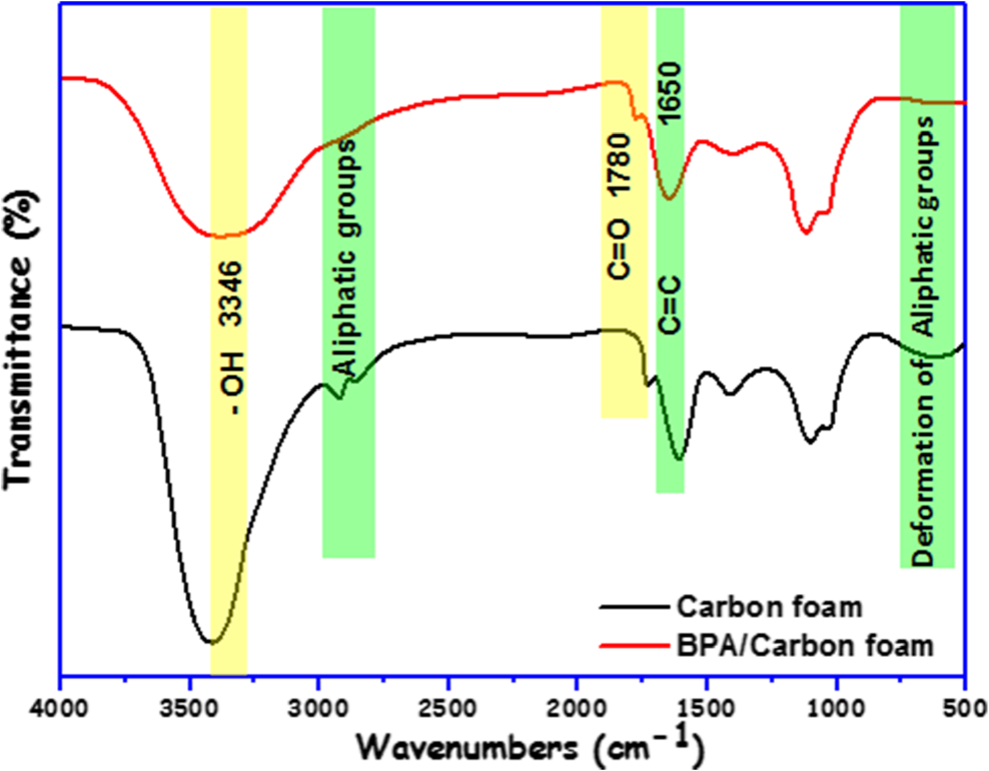
FTIR spectra before and after adsorption of BPA onto carbon foam

The results of Fourier transformed infrared (FTIR) investigation of the prepared carbon foam are presented in Fig. 8. The broadband at 3397 1/cm is related to the stretching vibration mode of the O–H groups (Carboxylic groups, phenyl groups, or adsorbed water) on the carbon foam surface (Sajjadi et al. 2018). The stretching frequency at 2922 and 2853 1/cm endorses the presence of CH_3_, CH_2_, and CH groups. The bands positioned at 1736 and 1604 1/cm were ascribed to the C=O in the carboxyl and lactonic groups and the C=C double bond (Zbair et al. 2018a, 2018c). The band at 1408 1/cm is associated with the aromatic C–C stretching. The peaks located at 1101 and 1023 1/cm were typical of various C–O and O–C–O stretching vibrations (Ahmed and Hameed 2018). The existence of C–H groups is disclosed with the band at 601 1/cm. This examination demonstrated the capability of carbon foam application as an adsorbent, due to the existence of potential uptake sites (Marques et al. 2018). By comparing the FTIR spectra of the carbon foam before and after BPA adsorption, some surface groups of the carbon foam structure were modified. The FTIR spectrum of the used carbon foam (BPA/Carbon foam) shows the disappearance of the aliphatic bands at 2922, 2853, and 601 1/cm. Moreover, the locations of the –OH, C=O, and C=C groups were shifted from 3397 to 3346 1/cm, from 1736 to 1780 1/cm, and from 1604 to 1650 1/cm, respectively. These changes may signify the hydrogen bonding formed between the –OH of BPA and –OH of the carbon foam. The change of the position of the C=O vibration revealed the existence of π–π interaction: According to Mattson et al. (Mattson et al. 1969), in π–π interaction, the C=O on the surface of the carbon foam acts as an electron donor and the aromatic rings of BPA act as electron acceptors. As indicated by the changed position of C=O, π–π interactions also play a role in the adsorption.

### Thermodynamic analysis

To define the thermodynamic information linked to the BPA adsorption onto the carbon foam, Gibbs’ free energy change (*ΔG*°, kJ/mol), entropy change (*ΔS*°, J/mol K), and enthalpy change (*ΔH*°, kJ/mol) were calculated using equations presented in Table 1S and the outcomes were summarized in Table 4. The negative values of *∆G*° (− 281.38 kJ/mol, − 28.08 kJ/mol, and − 28.023 kJ/mol) suggest that the BPA adsorption was a spontaneous and favorable process at all the used temperatures. Likewise, *∆S*° (26.7 J/mol K) has a positive value, which shows that the BPA molecules are arbitrarily dispersed on the surface of the carbon foam. The estimation of *∆H*°, – 4.8 kJ/mol, was consistent with the physisorption of BPA molecules on the carbon foam (Cardoso et al. 2011). Also, the *∆H*° value shows that the BPA adsorption onto the carbon foam was exothermic.

**Table 4:** Thermodynamic parameters of the BPA adsorption

*ΔH* (kJ/mol)	*ΔS* (J/mol K)		*ΔG* (kJ/mol)		
			293 K	303 K	313 K
		kg (L/mol)	103870	69399	51592.7
− 4.8	26.7		− 281.38	− 28.08	− 28.23

## Conclusion

In this study, the argan nut shell (ANS) was successfully used as a raw material for producing a structured carbon foam. The prepared foam structure presented a well-developed porous structure with different pore sizes and it had a specific surface area of 435 m^2^/g. The carbon foam showed 93% removal of BPA in the used experimental conditions. It was also found that the maximum adsorption capacity of BPA on the carbon foam according to the Liu isotherm (*Q*_Liu_) was 323.0 mg/g at 20 °C. Comparison of this value to the values of the other adsorbents demonstrates a high adsorption capacity of the developed carbon foam. The BPA adsorption kinetics on the carbon foam was best explained by the Avrami fractional model. Determination of thermodynamic parameters showed the adsorption to be exothermic and to take place via physisorption. To summarize, the structured carbon foam prepared from the argan nut shell is an excellent material for the BPA removal due to the following: (1) its easy preparation, (2) low price, (3) re-usability,(4) high adsorption capacity, and (4) facile separation from water, which makes it practical for real water purification applications.

## Electronic supplementary material

ESM 1 (DOCX 1454 kb)
