# Supplementary material for: Structured carbon foam derived from waste biomass: application to endocrine disruptor adsorption
Source: Environ Sci Pollut Res Int. 2019 Oct 19;26(31):32589–99. doi: 10.1007/s11356-019-06302-8 (PMC6875152; doi:10.1007/s11356-019-06302-8)
Supplement: Supplementary file 1 — (DOCX 1454 kb) [file 11356_2019_6302_MOESM1_ESM.docx]

# **Structured Carbon Foam Derived from Waste Biomass: Application to Endocrine Disruptor Adsorption**

**Mohamed Zbair^1,2^, Satu Ojala^2,*^, Hamza Khallok^3^, Kaisu Ainassaari^2^, Zouhair El Assal^2^, Zineb Hatim**^3^**, Riitta L. Keiski^2^, Mohamed Bensitel^1^, Rachid Brahmi^4^**

^1^Laboratory of Catalysis and Corrosion of Materials (LCCM), Department of Chemistry, Faculty of Sciences,

University of Chouaïb Doukkali El Jadida, BP 20, 24000 El Jadida, Morocco

^2^Faculty of Technology, Environmental and Chemical Engineering, University of Oulu, P. O. Box 4300, FI-90014 Oulu, Finland

^3^Team of Energy, Materials, and Environment, Department of Chemistry, Faculty of Sciences, University Chouaïb Doukkali, El Jadida, Morocco

^4^Laboratory of Coordination and Analytical Chemistry (LCCA), University Chouaïb

Doukkali, El Jadida -Morocco.

**Supplementary Information**

To whom correspondence must be addressed: [satu.ojala@oulu.fi](mailto:satu.ojala@oulu.fi)

**Table 1S. Equations used in this work to fit the data of BPA adsorption experiments.**

| **Equations & Models** | **Name** | **Parameters** | **Ref.** |
| --- | --- | --- | --- |
| $Q_{e,t}=\frac{{(C}_{0}{-C}_{e,t}) \times V}{m}$ | Adsorption capacity | C_0_ (mg/L) and C_e,t_ (mg/L) are the initial and equilibrium concentrations, respectively. m (g) is the weight of adsorbent and V (L) is the volume. | (Wang et al. 1998) |
| $Removal \%=\left( \frac{C_{0}{-C}_{e,t}}{C_{0}} \right) \times100$ | Removal efficiency | C_0_ (mg/L) and C_e,t_ (mg/L) are the initial and equilibrium concentrations. | (Garg et al. 2003) |
| $Q_{t}=Q_{\mathrm{cal}}(1-\exp^{K1t})$ | Pseudo-first-order kinetics | Q_cal_ and Q_t_ are the adsorbed amounts at equilibrium and at times t, respectively. K_1_: the rate constant. | (Tran et al. 2017) |
| $Q_{t}=\frac{(K_{2}Q_{\mathrm{cal}}^{2}t)}{(1+K_{2}Q_{\mathrm{cal}}t)}$ | Pseudo-second-order kinetics | K_2:_ rate constant | (McKay 1999; Tran et al. 2017) |
| $Q_{t}=Q_{e}(1-{\exp\left( - K_{\mathrm{AV}}. t \right)}^{n_{\mathrm{AV}}})$ | Avrami fractional-order kinetics | K_AV_ is the Avrami fractional-order constant rate (1/min), n_AV_ is a fractional kinetic order (Avrami), which is related to the adsorption mechanism. | (Lima et al. 2015) |
| $Q_{e}=\frac{Q_{L}K_{L}C_{e}}{1+K_{L}C_{e}}$ | Langmuir isotherm | K_L_: direct measure of the intensity of the adsorption process; Q_L_: maximum adsorption capacity. | (Langmuir 1916) |
| $Q_{e}=K_{f}C_{e}^{\frac{1}{n}}$ | Freundlich isotherm | K_F_: adsorption capacity; n: intensity of adsorption; | (Freundlich 1907) |
| $Q_{e}=\frac{Q_{\mathrm{Li}}.(K_{g}.C_{e})^{n_{L}}}{(1+{(K}_{g}.C_{e})^{n_{L}}}$ | Liu isotherm | Q_Li_ is the maximum sorption capacity of the adsorbent (mg/g)  K_g_ is the Liu equilibrium constant (L/mg)  n_L_ are the exponents of Liu model | (Saucier et al. 2015) |
| ${\Delta G}^{^{\circ}}=-RTlnK_{c}$ | Gibbs free energy | ΔG°: Gibbs free energy change; Kc: an equilibrium constant (dimensionless); R: gas constant; T: temperature. | (Anastopoulos and Kyzas 2016; Ghosal and Gupta 2017; Lima et al. 2019) |
| $\mathrm{Ln}K_{c}=\frac{{\Delta S}^{^{\circ}}}{R}-\frac{{\Delta H}^{^{\circ}}}{\mathrm{RT}}$ | Van’t Hoff equation | ΔS°: entropy change; ΔH°: enthalpy change. |  |


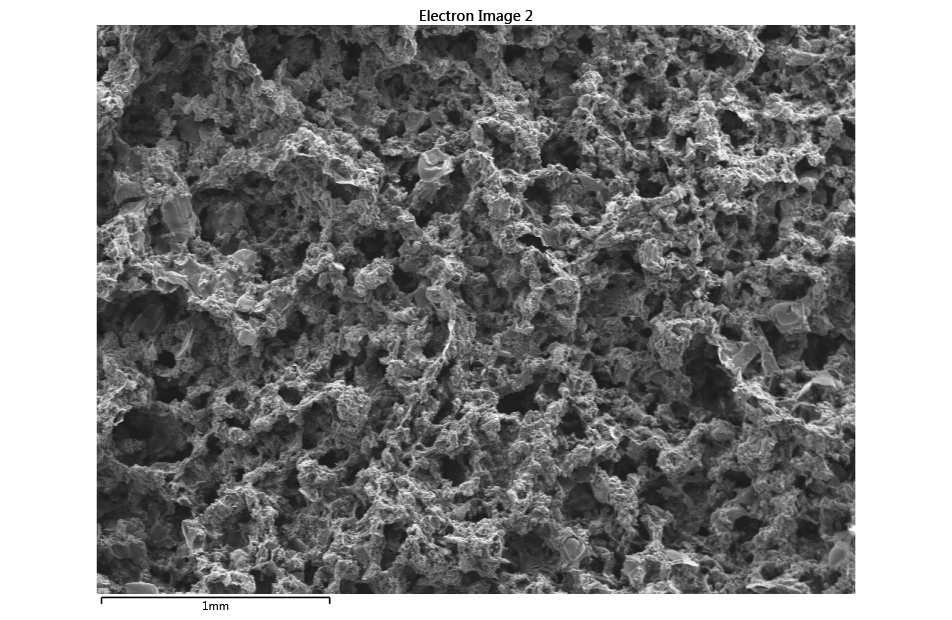


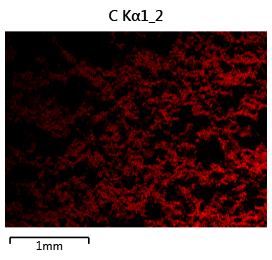

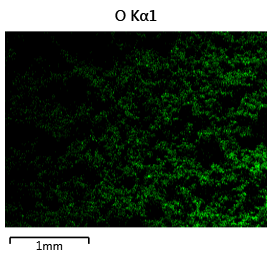

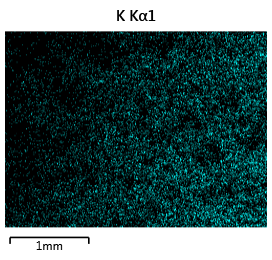

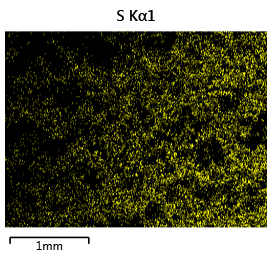

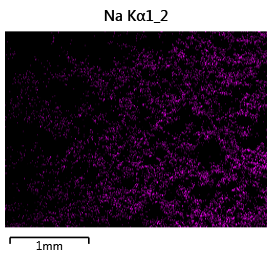


**Fig. 1S. FESEM image and mapping of the carbon foam.**

**References**

Anastopoulos I, Kyzas GZ (2016) Are the thermodynamic parameters correctly estimated in liquid-phase adsorption phenomena? J Mol Liq 218:174–185. doi: https://doi.org/10.1016/j.molliq.2016.02.059

Freundlich H (1907) Über die Adsorption in Lösungen. Zeitschrift für Phys Chemie 57U: doi: 10.1515/zpch-1907-5723

Garg VK, Gupta R, Yadav AB, Kumar R (2003) Dye removal from aqueous solution by adsorption on treated sawdust. Bioresour Technol 89:121–124. doi: 10.1016/S0960-8524(03)00058-0

Ghosal PS, Gupta AK (2017) Determination of thermodynamic parameters from Langmuir isotherm constant-revisited. J Mol Liq 225:137–146. doi: https://doi.org/10.1016/j.molliq.2016.11.058

Langmuir I (1916) The constitution and fundamental properties of solids and liquids. Part I. Solids. J Am Chem Soc 38:2221–2295. doi: 10.1021/ja02268a002

Lima ÉC, Adebayo MA, Machado FM (2015) Kinetic and Equilibrium Models of Adsorption. In: Bergmann CP, Machado FM (eds) Carbon Nanomaterials as Adsorbents for Environmental and Biological Applications. Springer International Publishing, Cham, pp 33–69

Lima EC, Hosseini-Bandegharaei A, Moreno-Piraján JC, Anastopoulos I (2019) A critical review of the estimation of the thermodynamic parameters on adsorption equilibria. Wrong use of equilibrium constant in the Van’t Hoof equation for calculation of thermodynamic parameters of adsorption. J Mol Liq 273:425–434. doi: https://doi.org/10.1016/j.molliq.2018.10.048

McKay G (1999) Pseudo-second order model for sorption processes. Proc Biochem 34:451

Saucier C, Adebayo MA, Lima EC, et al (2015) Comparison of a Homemade Bacuri Shell Activated Carbon With Carbon Nanotubes for Food Dye Removal. CLEAN – Soil, Air, Water 43:1389–1400. doi: 10.1002/clen.201400669

Tran HN, You SJ, Hosseini-Bandegharaei A, Chao HP (2017) Mistakes and inconsistencies regarding adsorption of contaminants from aqueous solutions: A critical review. Water Res 120:88–116. doi: 10.1016/j.watres.2017.04.014

Wang J, Huang CP, Allen HE, et al (1998) Adsorption characteristics of dye onto sludge particulates. J Colloid Interface Sci 208:518–528. doi: 10.1006/jcis.1998.5875
